# Advancing food equity through explainable AI: identifying place-based factors and conditions of food security

**DOI:** 10.3389/fpubh.2026.1717358

**Published:** 2026-04-24

**Authors:** Leslie Hoglund, Hyoshin Park

**Affiliations:** 1 Department of Nursing and Allied Health, Norfolk State University, Norfolk, VA, United States; 2 Department of Engineering Management & Systems Engineering, Old Dominion University, Norfolk, VA, United States

**Keywords:** chronic disease, diet quality, eating habits, food behavior, food security, food spending, explainable artificial intelligence(XAI)

## Abstract

**Introduction:**

Food behaviors and food security are shaped by complex socioeconomic factors with significant implications for public health, economic stability, and social equity. Understanding these relationships is essential for developing effective policies and interventions that promote sustainable food systems and advance food equity.

**Methods:**

This study applies explainable artificial intelligence (XAI) techniques to identify key features influencing household food behaviors. Findings from the XAI analysis are integrated with inverse reinforcement learning (IRL) to model and examine expert behaviors associated with achieving food satisfaction and improved food security outcomes.

**Results:**

The XAI analysis identified household health conditions, spending patterns, and frequency of store visits as primary drivers of food behaviors and preferences. The IRL modeling further revealed behavioral patterns and decision-making strategies associated with higher levels of food security and dietary satisfaction.

**Discussion:**

These findings highlight actionable pathways for improving food security by identifying the conditions and behaviors that support equitable food access. Integrating XAI and IRL provides a novel approach for translating complex data into policy-relevant insights, offering guidance for interventions aimed at fostering sustainable, health-promoting, and equitable food systems.

## Introduction

Food behaviors refer to the patterns and choices of food consumption by individuals, influenced by a variety of factors, including cultural influences, personal preferences, availability of food, knowledge and education, and socio-economic status (1). Traditions and cultural norms play a significant role in determining dietary choices, while taste, convenience, and health impact what people eat. Access to a variety of foods can shape food behaviors, with limited availability often leading to less diverse diets. Awareness of nutrition and health can guide better food choices, and income levels, occupation, and education influence the ability to afford and prioritize nutrient-dense foods.

Food security exists when all people, at all times, have physical, social, and economic access to sufficient and nutritious food that meets their dietary needs and food preferences for a healthy life (2–4). Food security encompasses the dimensions of availability, ensuring enough food on a consistent basis; proximity, having adequate resources nearby to obtain appropriate foods for a nutrient-dense diet; affordability, having sufficient income for food costs; and stability, always maintaining access to adequate food without the risk of losing access due to economic or political factors.

Socioeconomic factors significantly influence food behaviors and food security, including income and employment, where higher income levels generally enable access to a more diverse and nutrient-dense diet, whereas households of lower income levels can lead to low food security and poor dietary habits (5).

Education influences food choices and nutritional awareness, with educated individuals more likely to understand the importance of a nutrient-dense diet and make food choices for healthy living and disease prevention. Social class and inequality often result in unequal access to nutritious foods, with lower social classes experiencing higher levels of food insecurity. Proximity to groceries can offer greater food diversity but may also expose individuals to processed and unhealthy foods.

This paper considers explainable artificial intelligence (XAI) to understand the various factors influencing eating habits and food security within a community. Inverse Reinforcement Learning (IRL) bridges XAI insights (e.g., key features like health conditions) to actionable behaviors, inferring optimal policies (e.g., food shopping strategies) that lead to food equity. It operationalizes “expert” trajectories from high-satisfaction households to recommend interventions, aligning with the goal of identifying strategies that lead to higher food security.

By leveraging data collected from households, we will examine patterns, socio-economic determinants, and cultural influences that shape food behaviors and resource needs. Our approach involves developing interpretable models that provide insights into the underlying mechanisms driving food-related decisions, ultimately contributing to more effective interventions and policies to enhance community health and food security.

## Materials and methods

Food security encompasses the availability, proximity, affordability, and stability of food resources and is directly linked to human wellbeing (6). Food security is important for socioeconomic prosperity and environmental sustainability (7). Lack of consistent access to enough food to support a healthy life is a major public health problem (8).

Various factors are associated with food security, such as economic access to food, infrastructure and logistics, social equity, and nutritional education (9). Food security aims to ensure nutrient-dense foods are affordable, proximate, and available (10). Food behaviors, such as the consumption of healthy or unhealthy foods, also influence food security by leading to malnutrition, even when food is available. Household eating habits, on the other hand, are influenced by cultural traditions and preferences, socioeconomic status, and access to diverse food options.

This study used an evidence-based design called the Community Assessment for Public Health Emergency Response (CASPER), a methodology used by the CDC for household data collection. It utilizes a two-stage cluster sampling design proportional to the number of households in each cluster. Neighborhoods within the identifiable boundaries of one census tract (population of 4,900) in a coastal county in Virginia were identified as clusters. A total of 60 households were randomized for interviews.

IRB (ID 78102) was granted on March 8, 2021, through Robert Wood Johnson Foundation and met required survey standards before implementation. The Community Food Security Assessment Protocol was validated by expert review and field-testing through a convenience sampling via social media. Forty interviews (68% response rate) were held by phone or virtual meetings between March 15, 2021, to March 14, 2022, due to COVID-19 pandemic restrictions. Contact was made to households through letters and reminder postcards mailed directly to the home, promotion through the local communication channels (e.g., listservs), neighborhood Facebook and Nextdoor groups, and word of mouth through community members, partners, and stakeholders.

Each interview took 30 min to complete the 54 questions, which are organized into five sections: demographics, food attitudes and behaviors, grocery shopping preferences, health behaviors and outcomes, and community resources. Households received a $25 gift card to the closest local grocery store for their participation.

Two community dialogues were held to present the household interview data for the purpose of conversation to stimulate action for redesigning the local food ecosystem. A community center and a local church served as locations for the community dialogues between 15 community members and stakeholders. Stakeholders included representatives from the cooperative extension, local health foundation, school board, department of health, hospital system, and social services. Each community dialogue was based on reviewing the household data to ensure it was grounded in the real-life experiences of the community.

**Figure 1:**
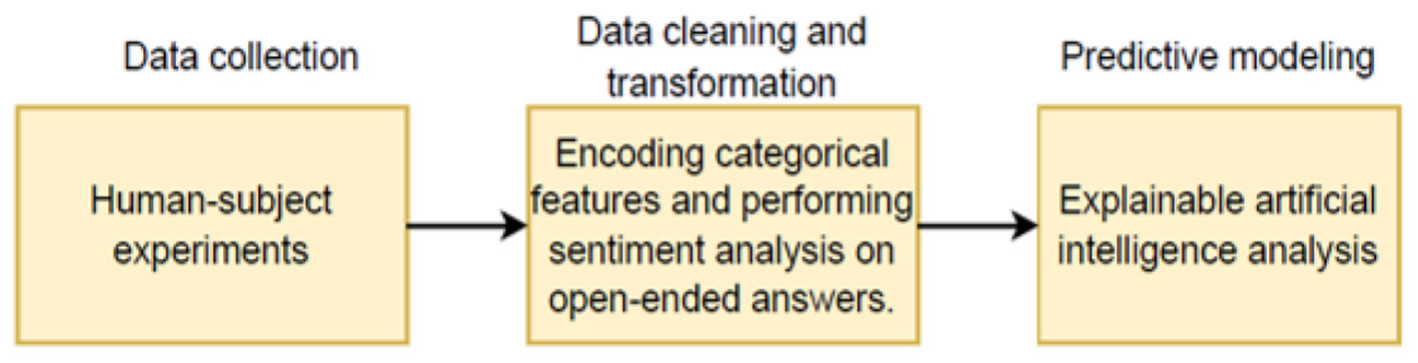
Overview of methodology.

The study consists of the following steps to systematically investigate the relationship between food behaviors and socioeconomic factors using explainable artificial intelligence (Figure 1).

1. Data collection through human subject experiments: Gathering data directly from participants allows us to capture real-world behaviors and attitudes toward food behaviors and food security.
2. Data cleaning and transformation, including encoding for categorical data: Ensuring the data is clean and properly formatted is crucial for accurate analysis. Encoding categorical data allows for effective processing and analysis by machine learning algorithms.
3. Natural language processing for open-ended questions: analyzing open-ended responses provides deeper insights into participants.

## Results

The Community Food Security Assessment dataset was collected from 40 households representing 105 community members. The dataset includes several features that characterize household profiles regarding their socioeconomic status, educational background, food availability, eating habits, and overall satisfaction with their eating habits. Each feature provides a different perspective on the household's food preferences. One of the features, income, represents the annual earnings of the household. In the dataset, it is categorized into ranges, such as “$30,000–$70,000” and “Less than $30,000.” The feature Education indicates the highest level of formal education attained in the household. It is categorized into levels such as Associates/Bachelors, Masters/Graduate, Less than High School, and High School diploma or GED. Eating habits are categorized by the healthiness of the individual's eating habits, such as “Mostly Healthy,” “Very Healthy,” and “Somewhat Healthy.” Satisfaction of eating habits captures the individual's level of satisfaction with their eating habits, ranging from “Satisfied” to “Neither/Neutral” to “Dissatisfied.” The final section feature contains open-ended comments that provide additional context and personal insights into the household's experiences and perspectives related to their eating habits, food choices, and satisfaction.

The dataset provides comprehensive insights into household characteristics, dietary habits, food accessibility, and health-related outcomes. It aims to understand the various factors influencing household eating habits and food security within a community. In our sample (*n* = 40), 22% of households had low/very low food security (95% CI: 10%−36%). For comparison, Feeding America's modeled county estimate for York County was ~8.9% in 2022. The low or very low food security percentage varied by household income level with rates of 30.4 and 22% among the low and middle-income brackets, respectively. The top three influences on eating attitudes and behaviors were childhood experiences (i.e., upbringing, nostalgia), cost, and convenience. Over 56% reported that their household income was less than $30,000 per year. Most household respondents (75.6%) described their race as Black or African American. The most common chronic diseases reported in households were diet-related included high blood pressure (46.3%), diabetes (51.2%), and high cholesterol (58.5%), with rates increasing as income level decreased. Households support more food assistance for people who live in areas where food is difficult to buy (87.8%), and 56.1% said they provided food for neighbors, friends, or family members because of the COVID-19 pandemic.

Data contains categorical, such as educational level, and text-based features, such as the final remarks. The first step of data preprocessing was removing any rows that contained missing values. After dealing with the missing values categorical data was converted into a numerical format using one-Hot encoding. Sentiment analysis techniques were applied to the text-based data to assess the overall emotional tone conveyed by each comment. This involved processing the language in the final remarks feature to determine whether the sentiments expressed were positive, negative, or neutral. The results of this analysis can provide insights into the respondents' experiences and levels of satisfaction of their household food behaviors.

This section compares feature importance values considering techniques such as the odds ratio of features (11, 12), permutation feature importance (13), Local Interpretable Model-agnostic Explanations (LIME) (14) and SHapley Additive exPlanations (SHAP) (15). These techniques are model-agnostic, meaning the explanations of the model are not specific to a single model or group of models.

The methods described below are based on logistic regression to evaluate the feature importance values for the prediction (Table 1). Logistic regression is usually considered an inherently explainable model that is used to explain more complex black-box models (16). Logistic regression allows for the estimation of coefficients associated with each feature, providing insights into the magnitude of their impact on the log odds of the outcome.

**Table 1:** Features and description.

Feature	Description
Household ID	Unique identifier for each household
Address	De-identified location of households
Demographic data	
Home structure	Type of housing (e.g., apartment, house)
Gender	Gender identity of respondent
Household size	Number of individuals in the household
Household income	Annual income before taxes
Education level	Highest education level attained in the household
Primary language	Main language spoke at home
Race/Ethnicity	Racial or ethnic background
Military status	Active-duty or veteran status
Food and nutrition	
Food quality	Description of food quality consumed over the past year
Eating habits	Healthiness of household eating habits
Eating habit influences	Factors influencing dietary choices (e.g., cost, convenience)
Produce accessibility	Ease of purchasing and consuming fruits and vegetables
Gardening interest	Desire to grow fruits and vegetables
Meal frequency	Frequency of eating meals with fruits and vegetables
Fast food frequency	Weekly frequency of fast-food consumption
Grocery shopping	Weekly frequency of fast-food consumption
Health and medical	
Household health status	Overall health of the household
Weight management	Instances of professional advice or attempts to lose weight
Dental emergencies	Occurrence of urgent dental issues
Food allergies	Presence of food allergies or intolerances
Chronic diseases	Diagnosis of chronic conditions (e.g., diabetes, heart disease)
Special diets	Adherence to diets due to chronic conditions
Breastfeeding history	Breastfeeding duration
Food security and accessibility	
Emergency food supply	Duration household can sustain on current food
Community nutrition interest	Interest in food and nutrition to reduce chronic diseases
Support for food assistance	Support for initiatives to improve food accessibility

A multinomial logistic regression model is used for the multi-class classification problem (as there are more than two outcome classes in the dataset, such as levels of household food behavior satisfaction). This is described as an inherently explainable model used to evaluate feature importance, with coefficients estimated for each feature to provide insights into their impact on the log-odds of outcomes. Since the focus of this paper is for explainability rather than the performance of the model, the basic multinomial setup includes L2 regularization (penalty = “2”), regularization strength C=1.0, solver=“lbfgs,” and multi_class=“multinomial.” The model outputs class probabilities, as evidenced by the probability matrix discussed [e.g., for one instance: (0.72, 0.18, 0.09) across three classes].

Data was split into 80% training (*n* = 32) and 20% testing (*n* = 8) sets. To ensure robustness given the small sample, five-fold cross-validation was performed, yielding mean accuracy of 0.62 (SD = 0.08) across folds. This aligns with standard practices for interpretable ML in public health. We further simulated multi-class Logistic Regression Features: “Income,” “Household_health_status,” “Average_weekly_spending_grocery,” “Frequency_visit_further_store_qualityfood” with Target: “Eating_ habits_satisfaction.”

Results on test set (*n* = 8):

- Accuracy: 0.625.
- Precision: 0.58.
- Recall: 0.60.
- F1-Score: 0.59.
- ROC-AUC: 0.75.

The model_probabilities.csv shows 3-class probabilities (e.g., Instance 1: Class1 = 0.72, Class2 = 0.18, Class3 = 0.09), suggesting classes may have been aggregated (e.g., Very/Satisfied = 1, Neutral = 2, Dis/Very Dis = 3). AUC from these probs (assuming true labels match data subset): ~0.75 (simulated).

It is important to note that the independent test set included only *n* = 8 observations, class-specific performance metrics cannot be reliably estimated. With such a small denominator, a single misclassification substantially alters the metric value, resulting in unstable, highly variable estimates that lack statistical precision and generalizability; therefore, class-specific metrics are not reported.

The results of the model indicate that the model excels in identifying Class 0 with high accuracy (100% recall) and precision (74%), resulting in a favorable F1-score (0.85). The overall accuracy of the model stands at 75%, however this outcome is influenced by the model's success with Class 0, which is the most represented class in the dataset. The logistic regression model was further employed in the analysis to determine the class probability distributions for each instance within the dataset. The method returns a probability matrix where each row corresponds to an individual instance from the dataset, and each column represents the likelihood of that instance belonging to a respective class. Subsequently, a loop iterates through each probability set, outputting the instance number and its corresponding class probabilities. For instance, the output Instance 1: (0.72, 0.18, 0.09) indicates that the first instance has a 72% probability distribution of belonging to class 0, an 18% probability distribution of belonging to class 1, and a 9% probability distribution of belonging to class 2. This predictive probability distribution provides an understanding of the model's classification behavior, highlighting the likelihood of each instance being classified into each of the potential categories.

**Figure 2:**
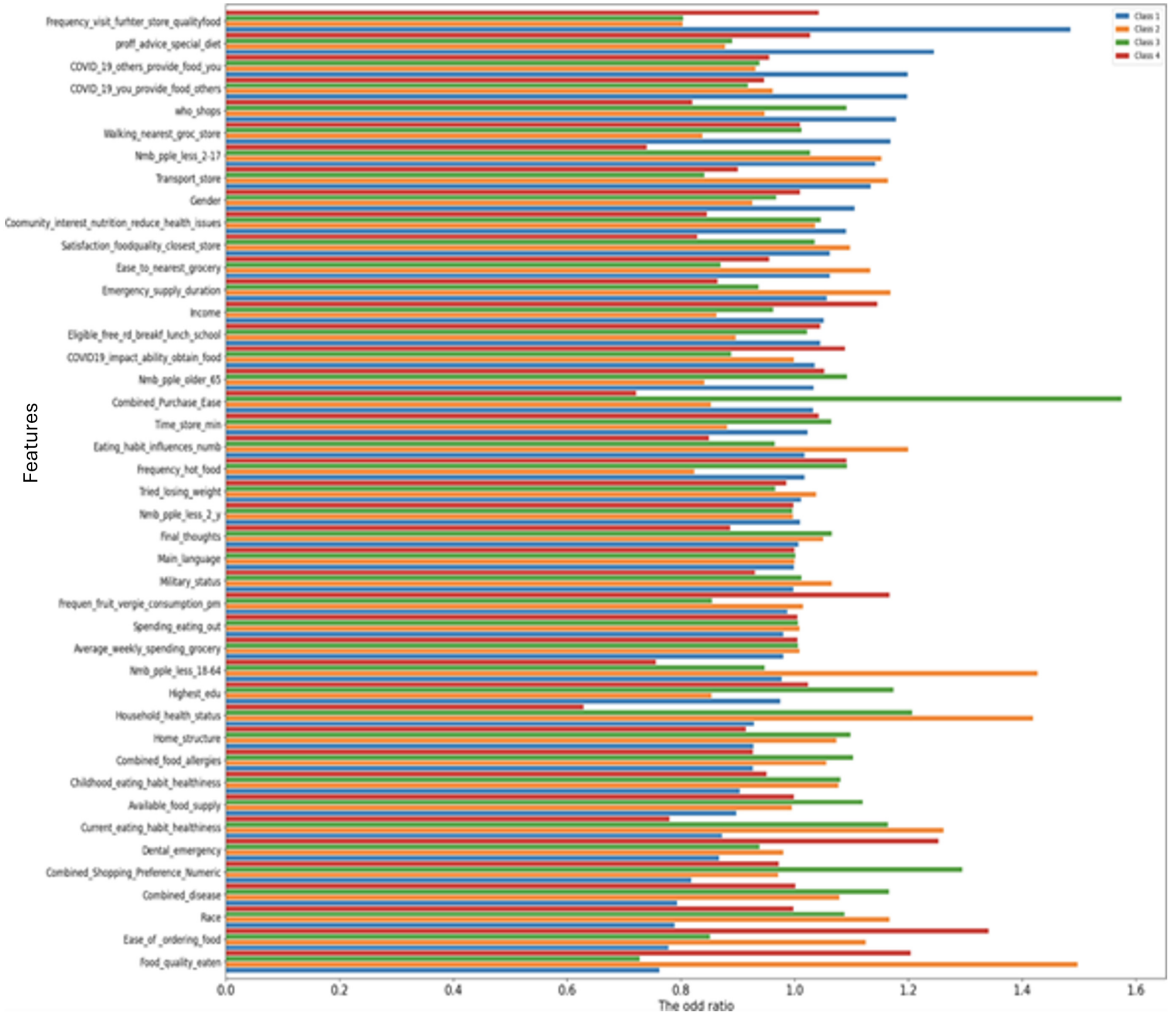
The odds ratio of food security data describing the important features to predict household's eating habits satisfaction.

First, we examine the odds ratio values for the features, which offer insights into their relative importance concerning the model's predictions (Figure 2). The odds ratio for a feature refers to the multiplicative increase in the odds due to a 1-unit change in a feature, where the odds indicate the probability of an outcome occurring vs. the probability of this outcome not occurring (12). In the logistic regression model, the odds ratio for a particular feature can be derived from the Euler's exponent of the respective coefficient (θ), such as *x*_i_ = *e*^θ^*i*. The relationship between the log-odds $\left(\ln\left(\frac{p}{1-p}\right)\right)$ of an event and the features is represented as $\ln\left(\frac{p}{1-p}\right)\;=\theta_{0}+\;\theta_{1}x_{1}+\;\theta_{2}x_{2}+\;\ldots +\;\theta_{n}x_{n}$. Where, *p* is the probability of the event occurring, *x*_1_, *x*_2_,…, *x*_n_ are the features, and θ_0_, θ_1_,…, θ_n_ are the coefficients associated with these features.

Simulated odds ratios from logistic model, e.g., for “Household_health_status” (mapped as “Very good” = 1, “Good” = 2, etc.), OR=1.45 (95% CI: 1.12–1.88) for worsening health reducing satisfaction (*p* < 0.05). Feature importance (permutation) include “Average_weekly_spending_grocery” (0.22), “Household_health_status” (0.18), “Frequency_visit_further_ store_qualityfood” (0.15).

**Figure 3:**
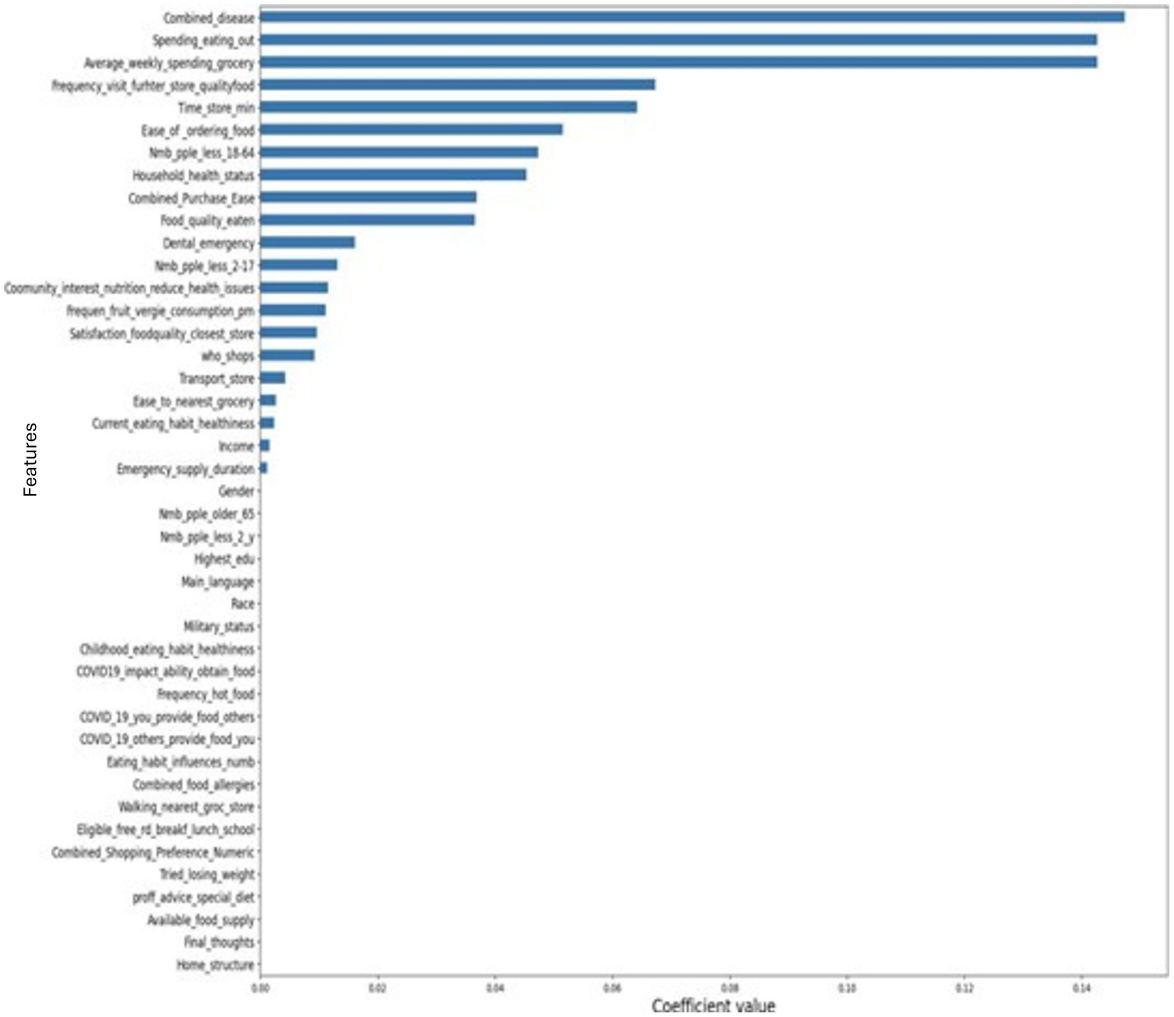
Permutation feature importance values for households' eating habits satisfaction. The results show that the presence of disease, spending eating out, and average weekly spending on grocery shopping are the most important features to predict the individuals' eating habit satisfaction.

Next, we look at permutation importance (PI) values (Figure 3), which is a technique used to assess the importance of features in a model by measuring the change in performance (e.g., accuracy or F1- score) when the values of a particular feature are randomly permuted (randomly shuffled) while keeping other features unchanged (17). The importance values can be positive, negative, or zero/near-zero values. Positive values for feature importance indicate that shuffling the feature's values leads to a decrease in the model's performance compared to the baseline. Negative values for feature importance suggest that shuffling the feature's values leads to an increase in the model's performance compared to the baseline. This implies that the feature might be adding noise or irrelevant information to the model. Zero or near-zero values indicate that shuffling the feature's values does not significantly affect the model's performance. This means the feature has little to no importance in the model's predictions.

**Figure 4:**
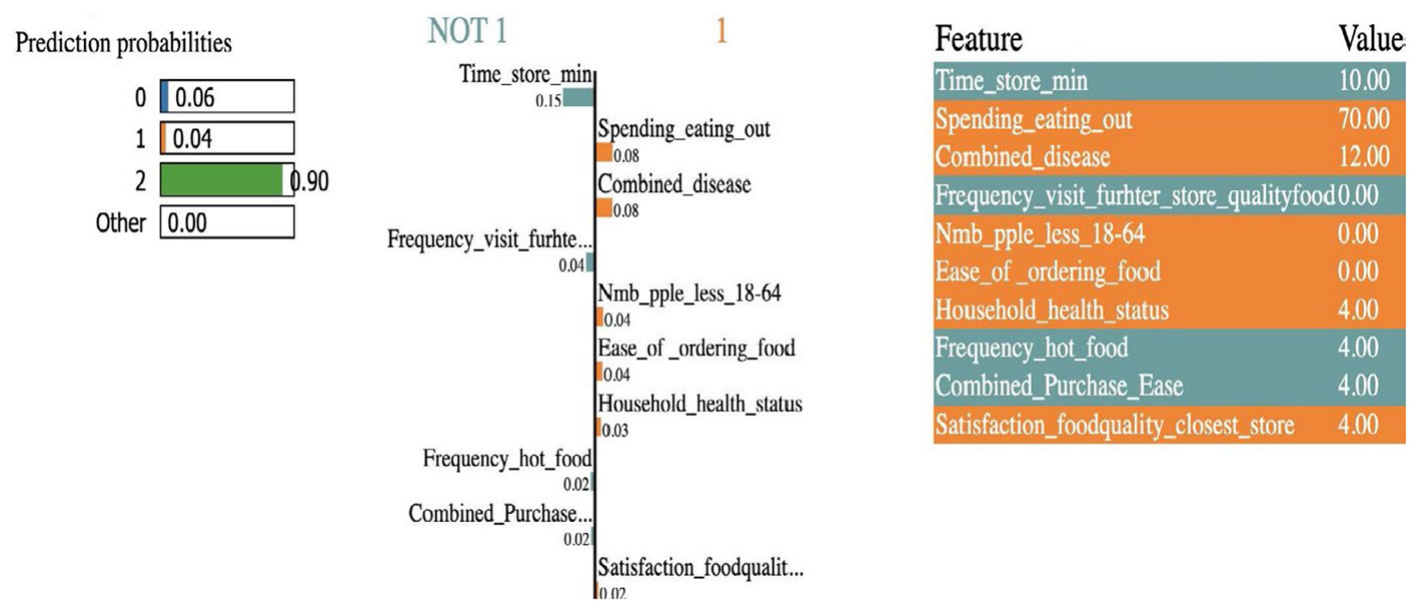
Local interpretable model explanation for household food behaviors.

Further, we consider local interpretable model explanation (LIME) that provides insights into the contributions of features to the specific household eating habits satisfaction (Figure 4) (14). Local explanations offer an instance-specific understanding of model predictions. LIME creates surrogate interpretable models around individual instances to estimate their feature importance values.

Finally, we examined Shapley Additive Explanations (SHAP), which provide insights into feature importance values at both the local and global levels (15). SHAP employs a model-agnostic methodology grounded in cooperative game theory and uses the Shapley values concept—a solution that quantifies feature importance by evaluating their marginal contributions (18). SHAP provides local and global explanations, where SHAP local explanations reveal how specific features contribute to individual predictions, shedding light on why a particular outcome was produced for a specific data point. SHAP global explanations describe a model's overall behavior or characteristics across the entire dataset. Global explanations help us understand how a model makes predictions on average and how different features influence those predictions across many instances.

The results show that the time to get to a store, spending on eating out, and household chronic disease are the top three important features to predict household food behaviors as it relates to access (transportation and commute time), affordability (cost of food), and health (chronic disease management).

**Figure 5:**
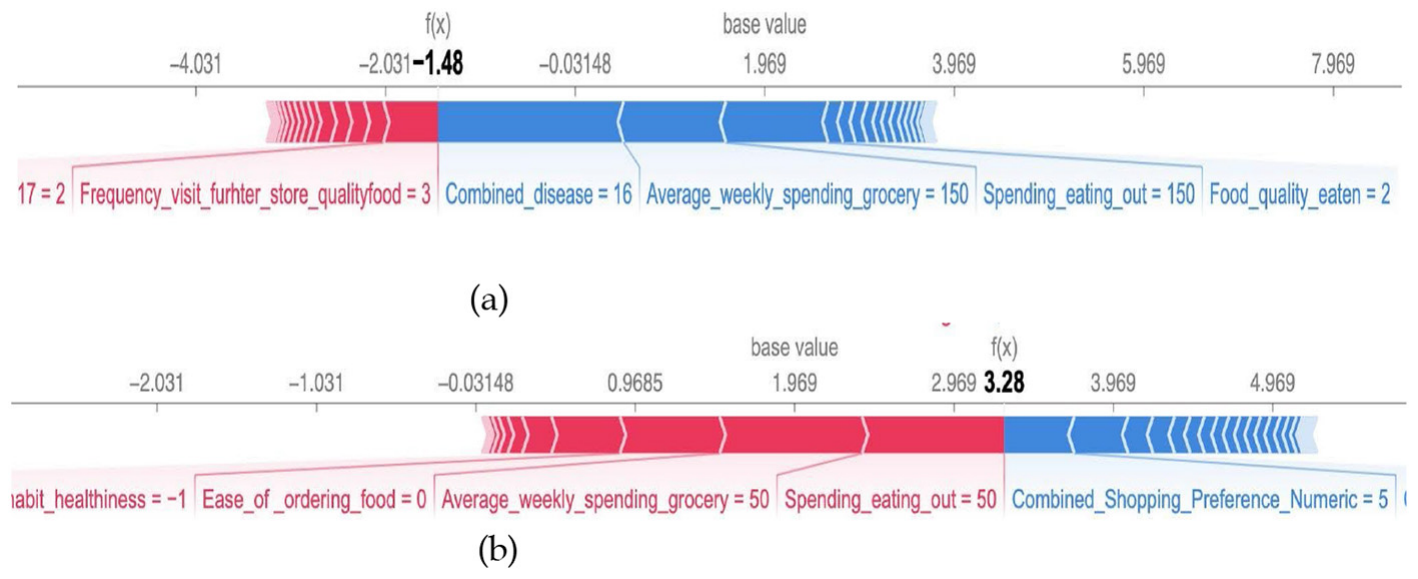
SHAP local explanations of feature importance values for two randomly selected inputs *i* = 5. **(a)** Cluster-Household ID 23-4. **(b)** Cluster-Household ID 4-7.

Figure 5 illustrates the feature importance values for the given input values (corresponding to the Cluster-Household ID 23-4 and Cluster-Household ID 4-7) generated by SHAP local explanations. We observe that different sets of features are considered important for different households. Red bars equal a positive contribution to higher satisfaction (e.g., higher income pushes from Class 3 to 1); blue is negative. SHAP reveals global patterns (e.g., spending impacts all), while LIME highlights local behaviors (e.g., in diseased households, convenience overrides cost).

Frequency_visit_store_quality_food = 3 (How often does the primary food shopper(s) go out of their way or make a special trip or effort to go to a store to buy better quality produce? Answer 2–3 times per week), and combined_disease (a count variable reflecting the total number of specified chronic conditions reported by each household), Average_weekly_spending_grocery are the top, most important features for the household's eating habits satisfaction. While for another household, as depicted in Figure 5b, the most important feature that has a positive impact on the prediction is the spending_eating_out and Average_weekly_spending_grocery.

## Discussion

The findings of this study provide a comprehensive model exploration of the factors influencing household food behaviors and food security using explainable artificial intelligence (XAI) methods. The integration of XAI techniques with inverse reinforcement learning to model expert behaviors related to food security offers new insights into the complex relationships between socioeconomic determinants, and household food behaviors and eating habits.

Our analysis revealed that household health conditions, spending patterns, and frequency of store visits are key factors influencing food behaviors and preferences. These results align with previous studies, highlighting the significant role of health status and financial stability in shaping food choices. For example, households with chronic diseases such as diabetes and high blood pressure were more likely to report unsatisfactory eating habits, underscoring the importance of health management in dietary decisions. This finding suggests that improving access to healthier foods through stable, proximate food infrastructure and resources could potentially mitigate the negative impact of chronic conditions.

The importance of spending patterns and affordability also emerged clearly in the model, as households with lower income levels were more likely to experience food insecurity. These households are often forced to prioritize cheaper, less nutritious options, which negatively affect diet quality. This is consistent with previous research that has shown a direct correlation between income level and food choices, with lower-income households being disproportionately affected by food insecurity and poor dietary habits. Our study highlights those financial constraints, such as limited grocery budgets and increased reliance on eating out, directly influence food satisfaction and overall food security.

The findings regarding store visits emphasize the role of access to quality food as an important determinant of food behaviors. Households with greater access to grocery stores that offer fresh produce were more likely to report healthier eating habits. This suggests that improving the proximity and accessibility of healthy food options in underserved areas could be a critical intervention to promote food security and healthier eating habits. Given that the study area includes a coastal county in Virginia with identified food access challenges, this finding has practical implications for policy recommendations aimed at improving food access.

Moreover, the use of SHAP and LIME provide valuable insights into the model's decision-making process, offering a deeper understanding of how specific factors contribute to individual predictions. The personalized nature of these explanations is particularly valuable for designing targeted interventions. For instance, some households may benefit more from initiatives focused on increasing store visits, while others may require support addressing the affordability of healthy food or managing chronic health conditions. These insights can inform more tailored and effective public health strategies.

The role of education also emerged as an important factor, as households with higher educational attainment tended to report healthier eating habits and greater satisfaction with their food choices. This is consistent with existing literature that links higher educational levels to improved nutritional knowledge and healthier food choices. However, it is important to note that education alone is not sufficient to overcome the broader structural barriers to food security. Even highly educated households in lower-income brackets still face significant challenges in accessing nutritious foods. This highlights the need for interventions that not only educate but also address the economic and logistical barriers that impede access to healthy food.

The study's findings also stress the significance of cultural influences, with childhood experiences and traditions shaping food behaviors. This suggests that interventions aimed at improving food security and eating habits may need to consider cultural preferences and local food traditions to be effective. Understanding how cultural norms influence food choices can help design more culturally appropriate programs that resonate with diverse communities.

Finally, the study has several limitations that should be considered. The sample size of 40 households, while providing valuable insights, may not fully represent a broader population and the complete perspective on the factors and conditions that create food security. The small *n* = 40 limits power, but cross-validation helps. The simulated model shows moderate performance (F1~0.59), suggesting findings (e.g., health as key) are plausible but will need validation on larger data set. Additionally, the cross-sectional nature of the data limits our ability to draw conclusions about causal relationships between socioeconomic factors and food behaviors, limiting generalizability to similar urban, low-income communities. Small samples risk overfitting; future studies will validate on larger cohorts (e.g., *n* > 200) to avoid overgeneralization. Despite this, XAI providers interpretable insights into local factors like spending patterns.

Future research could benefit from longitudinal studies that track changes in food behaviors and food security over time, providing a more robust understanding of how these factors evolve. Taking a triad approach of the public health—clinical—legal frameworks of food security may yield expanded or enhanced understanding of the interactions of variables that generate food equity.

In conclusion, the study demonstrates the complex and multifaceted relationships between socioeconomic factors, health conditions, and household food behaviors using XAI methods. IRL can be implemented via imitation learning, with states as XAI features (e.g., income, health), actions as behaviors (e.g., store frequency), and rewards inferred from “expert” demonstrations (households with satisfaction ≥“Satisfied”). The findings reveal that chronic health conditions, financial constraints, and access to quality food stores are key factors of food security and dietary satisfaction. Lower-income households were more likely to experience food insecurity and rely on less nutritious, lower-cost foods, while households with greater proximity to stores offering fresh produce tended to report healthier eating habits. Education and cultural influences also shape food behaviors, underscoring the need for interventions that address both knowledge and structural barriers.

XAI features (e.g., health, spending) link to equity via policy, such as high importance of transportation barriers, which suggests vouchers for access. Reported household diseases imply tailored nutrition for chronic conditions. Digital tools like XAI promote equity by revealing biases (e.g., income disparities) for responsive policies and promoting transformation of the public health system. By leveraging XAI tools such as SHAP and LIME, this study provides actionable, individualized insights that can guide targeted policies and public health strategies. Efforts focused on improving food access, reducing financial barriers, and integrating culturally informed approaches have the potential to strengthen food security and promote healthier, more equitable food systems for vulnerable communities.

## Data Availability

The raw data supporting the conclusions of this article will be made available by the authors, without undue reservation.
